# The Migration, Diversity, and Evolution of *Puccinia triticina* in China

**DOI:** 10.3390/plants13172438

**Published:** 2024-08-31

**Authors:** Lin Zhang, Panpan Zhao, Qingfang Meng, Hongfei Yan, Daqun Liu

**Affiliations:** 1 Biological Control Center of Plant Diseases and Plant Pests of Hebei Province, College of Plant Protection, Hebei Agricultural University, Baoding 071000, China; zhanglin42@163.com (L.Z.); zpp3210@126.com (P.Z.); liudaqun@caas.cn (D.L.); 2 School of Landscape and Ecological Engineering, Hebei Engineering University, Handan 056038, China

**Keywords:** *Puccinia triticina*, virulence, EST-SSR, genetic diversity, spread

## Abstract

Wheat leaf rust, caused by *Puccinia triticina*, is one of the most common fungal diseases of wheat in China and occurs widely in various wheat-growing regions. To clarify the epidemic, spread rules, and population structure of *P. triticina* among different regions, 217 isolates of *P. triticina* collected from Hebei, Shandong, Sichuan, and Xinjiang in China were tested by 34 Thatcher near-isogenic lines and 21 pairs of EST-SSR primers. A total of 83 races were identified, and THTT, PHTT, THTS, and PHJT were the most predominant races in the four provinces in 2009. We found enriched virulence and genetic diversity in the four *P. triticina* populations and a significant correlation between genetic polymorphism and geographic regions. However, no significant correlation was found between virulence phenotypes and molecular genotypes. Moreover, a notable high level of gene flow (*N_m_* = 2.82 > 1) among four *P. triticina* populations was detected. The genetic relationship among Hebei, Shandong, and Sichuan populations was close, possibly due to the spread of *P. triticina* from Sichuan to Shandong and then to Hebei. In contrast, the Xinjiang population was relatively independent. Genetic differentiation analysis showed some level of differentiation among or within populations of *P. triticina* in the four provinces, and the genetic variation within populations (74.97%) was higher than across populations (25.03%). Our study provides a basis for a better understanding of the regional migration, epidemic, and population structure of *P. triticina* in China.

## 1. Introduction

*Puccinia triticina* Eriks., the wheat leaf rust fungus of common wheat (*Triticum aestivum* L.), is prevalent in wheat-growing regions worldwide [1]. Wheat leaf rust is well adapted to the climate of wheat growth, so it is more widely distributed than stem rust or stripe rust [2,3,4,5]. The occurrence and prevalence of leaf rust under favorable climatic conditions can cause significant yield losses in susceptible wheat cultivars [4]. Recent studies predicted that the annual average global food loss attributable to wheat leaf rust will be 8.6 million metric tons from 2000 to 2050 based on a conservative scenario and 18.3 million metric tons based on a high-loss scenario [6]. China is the largest wheat producer in the world, with over 23.63 million hectares of wheat-cultivation area and 136.59 million tons of yield in 2023 (National Bureau of Statistics of China). However, China is also one of the regions most severely impacted by wheat leaf rust, with notable epidemics occurring in 1969, 1973, 1975, 1979, 2012, 2013, 2015, and 2020, leading to serious yield loss in the major wheat-growing regions [7,8]. According to statistics, wheat leaf rust affects approximately 15 million hectares annually in China, resulting in a yield loss of approximately 3 million tons. Resistant cultivars, fungicides, or an improvement of disease management practices are the most commonly used methods to control the disease [6], among which the promotion and application of resistant cultivars is the most economical, effective, and environmentally friendly [9]. However, due to the high variation rate of *P. triticina*, new races constantly evolve and eventually overcome the resistance of wheat cultivars, threatening wheat production [6]. Therefore, studying the virulence dynamics and population genetic structure of *P. triticina* will significantly contribute to the control of the occurrence and development of the disease and also guide wheat disease resistance breeding programs.

Since the 1920s, race surveys and virulence identification of *P. triticina* have been carried out to monitor the virulence dynamics of *P. triticina* populations. The annual race and virulence survey of *P. triticina* in the United States began in 1926 and in Canada since 1931 [10,11,12,13]. The comprehensive and systematic analyses of the race and virulence dynamics of *P. triticina* were conducted in the United States from 2000 to 2020 [14] and in Canada from 1997 to 2010 [15,16,17]. Moreover, similar studies have been reported in France [18], South America [19], Ukraine [20], India [21,22], Egypt [23], and Iran [24]. In China, studies on the virulence dynamics of *P. triticina* races began in the 1940s [25]. Wheat-growing regions in China are mainly divided into North China, Huang-Huai-Hai, the middle and lower reaches of the Yangtze River, and southwest and northwest regions. In the past, wheat leaf rust mainly occurred in the Southwest China wheat-growing region. However, climate warming has triggered the spread of leaf rust to the north of China. In the 1990s, leaf rust was more severe in all the wheat-growing regions across the country, especially in the Huang-Huai-Hai wheat-growing region. Since 1986, regional race and virulence studies of *P. triticina* in China have been carried out based on the Thatcher near-isogenic lines [7,8,26,27,28,29,30,31]. These studies on population dynamics of *P. triticina* based on virulence are of great significance in studying the virulence variation and diversity of *P. triticina*. Unlike *P. striiformis* f. sp. *tritici*, the regional epidemics and spread patterns of *P. triticina* in China are currently unclear. *P. striiformis* mainly spread from southern Gansu and northwest Sichuan to other wheat-growing regions, and *P. striiformis* in the northwest of China, especially in Xinjiang, is different from other regions in terms of population structure and spread [32,33]. However, studies on the regional epidemics and spread rules of *P. triticina* are still rare and unsystematic, so it is necessary to strengthen the study of Chinese *P. triticina* in migration, spread, and population structure.

Molecular marker technologies have been widely applied to evaluate the genetic diversity of *P. triticina* populations in the past 20 years [34]. The development of these technologies enables us to understand the migration, evolution, diversity, and population structure of *P. triticina* at the molecular level. Random amplified polymorphic DNA (RAPD) [35,36,37], amplified fragment-length polymorphism (AFLP) [38], simple sequence repeat (SSR) [39], and expressed sequence tag-simple sequence repeat (EST-SSR) [40,41,42,43] have been successively developed and applied to study the population structure and genetic diversity of *P. triticina*. A previous study found that the *P. triticina* population of China was strongly differentiated from other regions of the world, which may indicate that the Chinese *P. triticina* population may be endemic in its own region [44]. However, there are few studies on the genetic diversity, migration, spread, and epidemic routes of *P. triticina* populations among different regions of China. Therefore, in order to clarify the relations of the population of different regions and the rules of the epidemic in different regions, this study analyzed the genetic and virulence diversity of *P. triticina* populations in Heibei Province, Shandong Province, Sichuan Province, and the Xinjiang Uygur Autonomous Region of China, which can provide further evidence for the regional spread and migration of *P. triticina* in China.

## 2. Results

### 2.1. Dynamics of Races

In total, 217 single-uredinial isolates were selected from Heibei (48 isolates, No. 1–48), Shandong (48 isolates, No. 49–96), Sichuan (44 isolates, No. 97–140), and Xinjiang (77 isolates, No. 141–217) in 2009 (Table 1). These isolates were successfully identified in 83 races (Table 1 and Table 2). THTT, PHTT, THTS, and PHJT were the predominant races with 23.50%, 9.22%, 8.29%, and 5.53% frequencies, respectively (Table 3). However, there were still slight differences in the types of predominant races among different regions (Table 2 and Table 3).

### 2.2. Virulence Polymorphism Analysis

**Figure 1:**
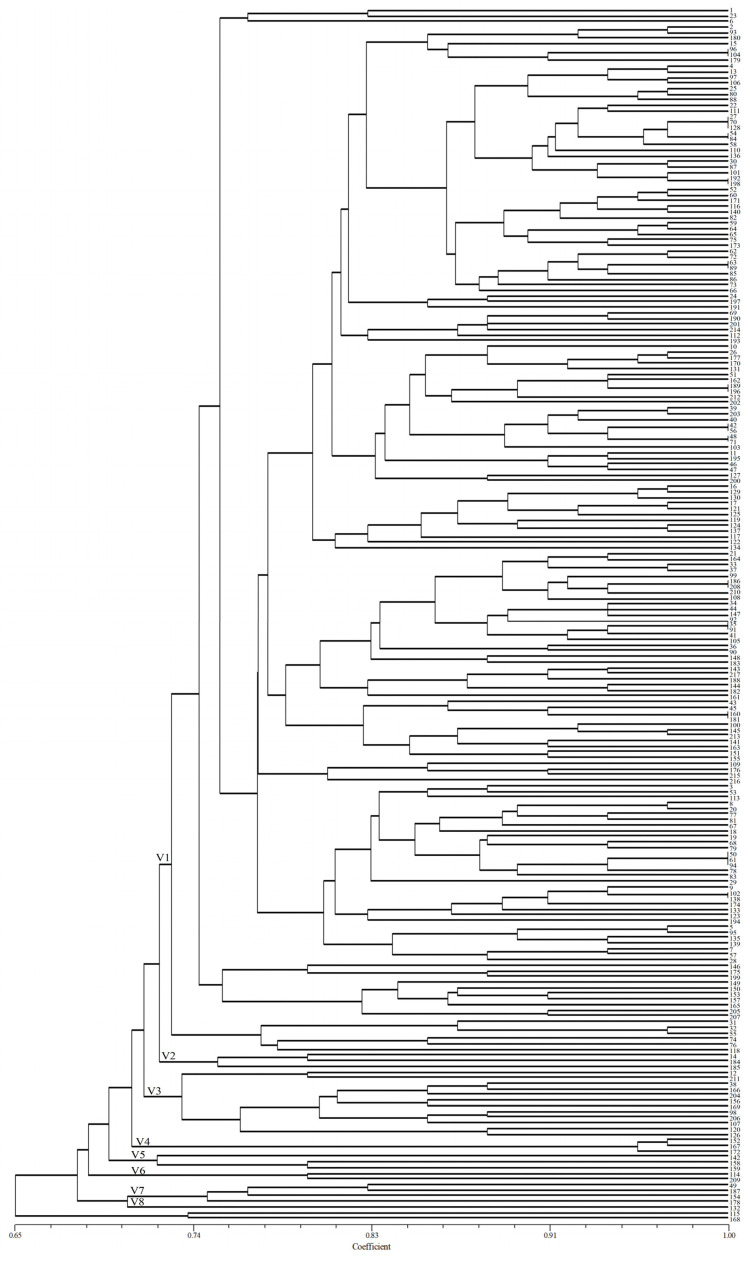
UPGMA dendrogram of *Puccinia triticina* based on virulence polymorphism.

The similarity coefficient of virulence between 217 isolates was 0.65–1.00 by constructing dendrograms of virulence based on the UPGMA method (Figure 1). All *P. triticina* isolates were clustered into nine groups (V1–V9) when the similarity coefficient was 0.73. A total of 187 isolates (86.18%) were grouped in cluster V1, and 30 isolates were spread across the other groups (V2–V9). The results showed that these isolates were not clustered into different groups according to their source regions, which indicated that the virulence polymorphism of *P. triticinia* populations was not closely correlated with geographical origin, although a few isolates with the same geographical origin were clustered in the same group. 

### 2.3. Genetic Polymorphism Analysis

Two hundred and seventeen isolates of *P. triticina* in 2009 were analyzed by 21 pairs of EST-SSR primers. Only 18 pairs of primers could amplify clear and stable polymorphic bands, and a total of 48 alleles were amplified, of which 30 alleles were polymorphism alleles, and the percentage of polymorphism was 62.50%. The number of alleles and polymorphic loci of these 18 pairs of primers was 2–4 and 1–3, respectively (Table 4).

**Figure 2:**
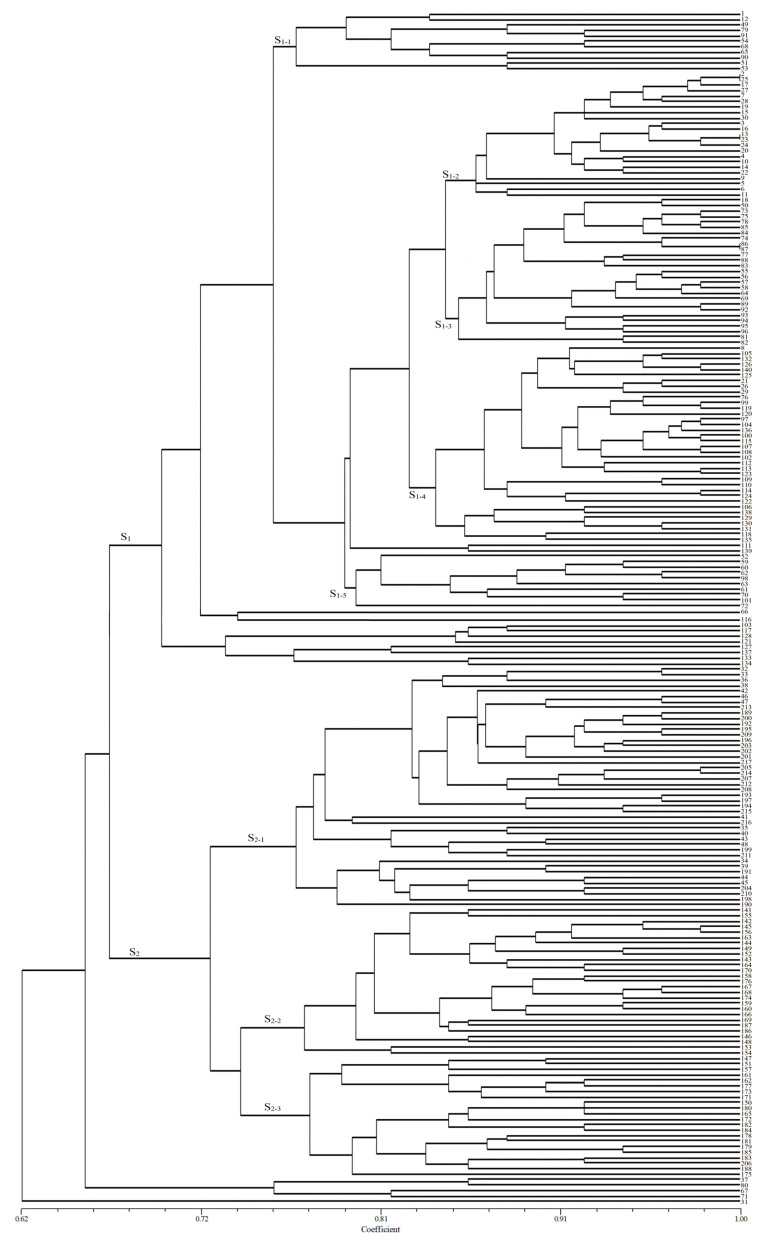
UPGMA dendrogram of *Puccinia triticina* based on EST-SSR polymorphism.

The genetic similarity coefficient based on an EST-SSR analysis of 217 isolates was 0.62–1.00 (Figure 2). Most isolates were partitioned into two main clusters (S1 and S2) at a similarity coefficient of 0.68. Cluster S1 contained most of the isolates from Hebei, Shandong, and Sichuan. These isolates were clustered into different subclusters according to the source region, such as subclusters S1-1, S1-3, and S1-5 which mainly contained the isolates of Shandong, subcluster S1-2 which mainly contained the isolates of Hebei, and subcluster S1-4 which mainly contained the isolates of Sichuan. Cluster S2 contained three subclusters, S2-1, S2-2, and S2-3, and most of the isolates of these subclusters were mainly from Xinjiang. These results showed that most of the isolates originating from the same region were clustered in the same group or several neighboring subclusters with a high level of similarity, indicating that the genetic structure in these isolates may be related to their source region. Moreover, the clustering results also showed that the isolates from Hebei, Shandong, and Sichuan were closely related, while the isolates from Xinjiang were far from the other three regions.

### 2.4. Correlation Analysis between Virulence Polymorphism and EST-SSR Polymorphism

By comparing the virulence polymorphism and EST-SSR polymorphism cluster analysis dendrogram of *P. triticina*, it was found that there was no obvious correlation between the genetic cluster based on EST-SSR and virulence characteristics of *P. triticina* isolates. The isolates with different phenotypes may have the same or similar DNA fingerprints and have close relationships with each other. Conversely, isolates with the same phenotype may have different DNA fingerprints and distant genetic relationships. In addition, the correlation coefficient of virulence and EST-SSR polymorphisms was 0.08 (<0.3) by using the MXCOMP module of NTSYS-pc, which also indicated that there was no correlation between virulence and EST-SSR polymorphism.

### 2.5. Two-Dimensional Principal Coordinate Analysis of EST-SSR and Virulence

**Figure 3:**
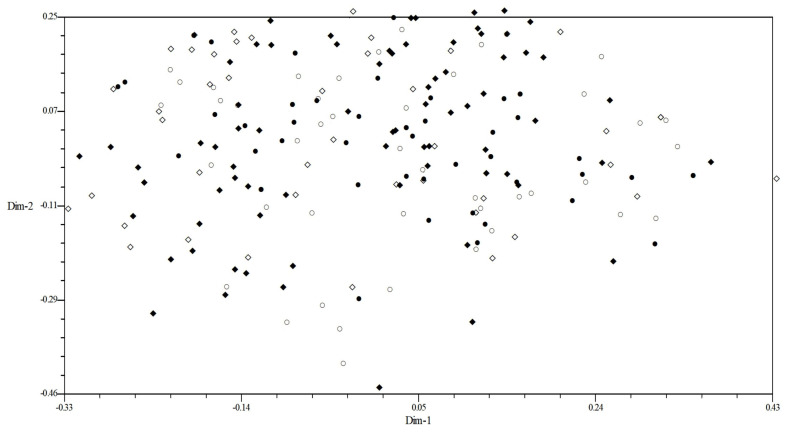
Two−dimensional plot of principal coordinates analysis of *Puccinia triticina* based on virulence polymorphism. Note: ○, Hebei; ●, Shandong; ◇, Sichuan; and ◆, Xinjiang.

**Figure 4:**
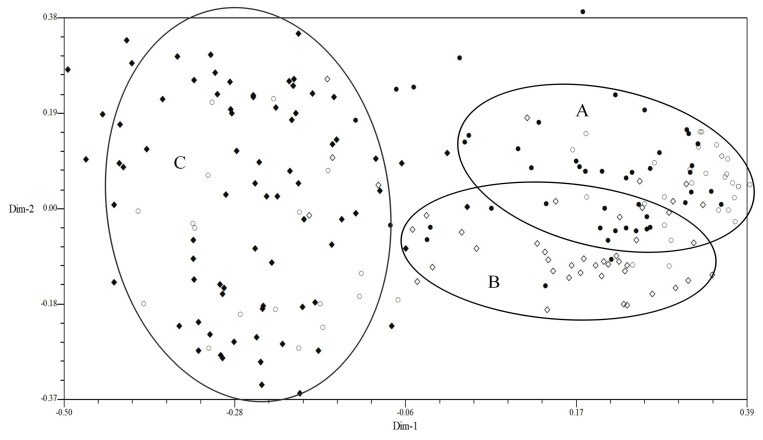
Two−dimensional plot of principal coordinates analysis of *Puccinia triticina* based on EST-SSR polymorphism. Note: ○, Hebei; ●, Shandong; ◇, Sichuan; and ◆, Xinjiang. Area A contains most isolates from Heibei and Shandong, area B contains most isolates from Sichuan, and area C contains most isolates from Xinjiang.

In order to further determine the correlation between virulence polymorphism or EST-SSR polymorphism and geographical region, all isolates were analyzed by a 2D-PCA plot analysis. In the 2D-PCA plot analysis of virulence, all isolates from four provinces were staggered without obvious regional distribution (Figure 3). In the 2D-PCA plot analysis of EST-SSR, all isolates were also mainly distributed in three areas (A–C) (Figure 4). The isolates from Heibei and Shandong were mainly concentrated in area A, which indicated that these isolates have similar genetic loci. In addition, a few isolates of Hebei were also distributed in area C. The isolates from Sichuan were mainly concentrated in area B, and isolates from Xinjiang were mainly concentrated in area C. These results showed that most of the isolates were obviously clustered according to the geographical region. The population in Xinjiang was relatively independent, while those in the other three regions were relatively close. This further confirmed that the molecular polymorphism based on the EST-SSR analysis was more closely related to the geographical region than the virulence phenotype polymorphism.

### 2.6. Virulence and EST-SSR Polymorphism Analysis of Predominant Races

**Figure 5:**
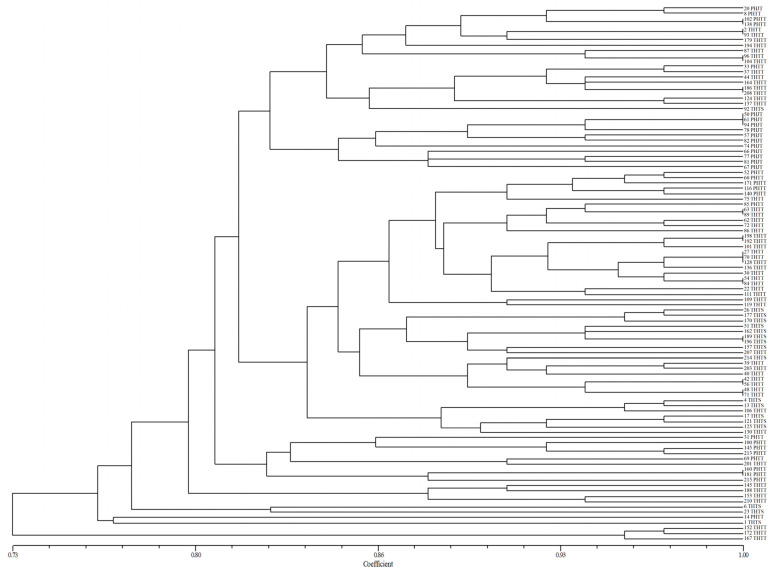
UPGMA dendrogram of *P. triticina* predominant pathotypes based on virulence.

**Figure 6:**
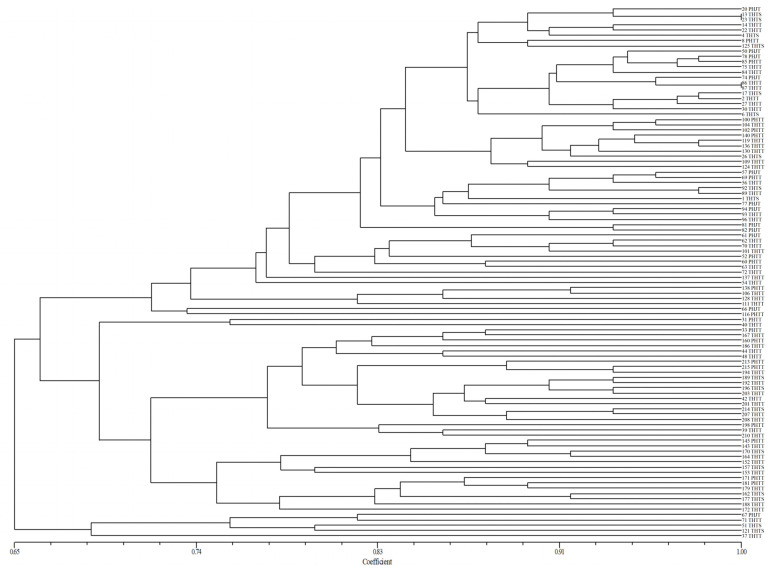
UPGMA dendrogram of *Puccinia triticina* predominant pathotypes based on EST-SSR.

In order to further determine whether the pathotypes were related to virulence polymorphism or EST-SSR polymorphism of *P. triticina*, a cluster analysis of 101 isolates of four predominant races (THTT, PHTT, THTS, and PHJT) was carried out based on virulence or EST-SSR. In the polymorphism analysis based on virulence, most of the same races tended to cluster in the same or adjacent cluster or subcluster (Figure 5), indicating a certain correlation between the pathotypes of the isolates and their virulence polymorphisms. In contrast, the clustering results of most races based on the EST-SSR polymorphism have no obvious rules (Figure 6). The isolates of different races may have the same or similar DNA fingerprints and be grouped into a cluster, while those of the same race with varying fingerprints of DNA can also be grouped into different clusters. The results showed no significant correlation between the phenotype and EST-SSR polymorphism.

### 2.7. Genetic Diversity Analysis of Puccinia triticina Population

All *P. triticina* isolates have been classified into four distinct populations based on their regions. Genetic diversity analysis among different populations was further conducted using the POPGEN version 1.32. The percentages of polymorphic loci were 83.67 in Hebei, 67.35 in Shandong, 71.43 in Sichuan, and 81.63 in Xinjiang (Table 5). Nei’s gene diversity index (*H*) and Shannon’s information index (*I*) both reflect the degree of variation among different populations with similar trends and the higher the value of H and I, the richer the corresponding gene variations [45]. Our results showed that the Nei’s gene diversity index (*H*) of each population within the *P. triticina* populations ranged from 0.28 to 0.36, and Shannon’s information index (*I*) ranged from 0.40 to 0.52, while the highest and lowest levels of genetic diversity were found in Heibei and Shandong, respectively (Table 5).

In addition, the analysis results of POPGEN software showed that the total genetic diversity (*Ht*) of all isolates, the genetic diversity within the population (*Hs*), and the genetic diversity among populations (*Dst*) were 0.35, 0.30, and 0.05, respectively. Moreover, the coefficient of genetic differentiation (*Gst*) was 0.15, of which genetic diversity within populations accounted for 85.71% of the total genetic diversity, and genetic diversity among populations accounted for 14.29%. There was also genetic differentiation among or within *P. triticina* populations per the AMOVA method of Arlequin ver. 3.11. Genetic variation within populations accounted for 74.97% of the total variation, while the genetic variation among the population accounted for 25.03% (Table 6), indicating that variations are mainly found within populations. Moreover, a relatively high level of gene flow (*Nm* = 2.82 > 1) was detected among the four populations. These results indicated a certain degree of exchange of inoculum sources across populations of different regions, which may play a critical role in keeping the similarity of populations’ genetic structure.

To further clarify the genetic relationship between different *P. triticina* populations from four regions, the populations’ genetic distance and genetic identities were estimated using the POPGENE software (Table 7), and the results showed that the population in Hebei had the closest genetic distance and the highest genetic identity to that in Shandong.

**Figure 7:**
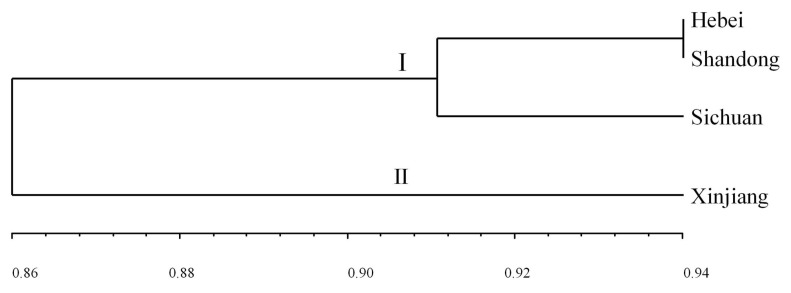
UPGMA dendrogram of *Puccinia triticina* populations based on EST-SSR data.

A dendrogram (UPGMA, unweighted average method) was then constructed by MEGA 7.0 software based on Nei’s genetic distances (Figure 7). The four populations could be clustered into two main branches (I–II) (Figure 7). The genetic relationship among the three populations from Hebei, Shandong, and Sichuan was relatively close, especially Hebei and Shandong, while the populations from Xinjiang had the farthest relationship with other populations. This indicated that the genetic relationship among populations of *P. triticina* had a certain correlation with geographical distribution.

## 3. Discussion

Wheat leaf rust is one of the most widespread fungal diseases in China and occurs in all wheat-growing regions across the country. Due to the lack of studies on the spread and migration of *P. triticina* in China, the regional epidemic of *P. triticina* is relatively unclear, especially compared with that of *P. striiformis* f. sp. *tritici*. So, to clarify the migration route, population diversity, and genetic variation of *P. triticina* in China, the method of virulence identification combined with EST-SSR molecular marker technology in this study was used for 217 *P. triticina* isolates from Hebei, Shandong, Sichuan, and Xinjiang in China. The results showed that the analysis methods can reveal the polymorphisms and differences in the genetic structure of *P. triticina* populations to a certain extent.

In this study, a relatively rich genetic diversity was found among the *P. triticina* isolates from Hebei, Shandong, Sichuan, and Xinjiang in China, as well as a difference in the genetic structure of *P. triticina* populations in different regions. In general, the main factors affecting the genetic structure of *P. triticina* populations are the mutation of the pathogen, the selection effects of host cultivars, and the climate environment. Mutation is likely a recurrent event in *P. triticina* populations because new virulence phenotypes are often detected shortly after the release of wheat cultivars with race-specific *Lr* genes [5,46]. So, some level of genetic diversity was maintained mainly by mutation in the highly clonal populations of *P. triticina* [47]. In the field populations of *P. triticina*, ultraviolet radiation accounts for most mutations [48]. According to the altitude, latitude, and climate of different regions in China, among the four provinces of Hebei, Shandong, Sichuan, and Xinjiang, the ultraviolet intensity of Sichuan is the weakest, while Xinjiang is the highest. Therefore, the mutation probability may differ in these regions, and the *P. triticina* isolates are regional specificity in Xinjiang. In addition, the genetic diversity of *P. triticina* can be affected by wheat cultivars and regional environments [49]. The four provinces of Hebei, Shandong, Sichuan, and Xinjiang belong to three different wheat-growing regions. Hebei and Shandong belong to the North China Plain wheat-growing region, Sichuan belongs to the southwest wheat-growing region, and Xinjiang belongs to the northwest wheat-growing region, and the wheat cultivars planted in these regions are quite different. For example, the main wheat cultivars in Sichuan are ‘Chuanmai’ series cultivars, ‘Xindong’ and ‘Xinchun’ series cultivars are the main wheat cultivars in Xinjiang, the main wheat cultivars in Shandong are ‘Jimai’, ‘Lumai’ and ‘Yanmai’ series cultivars, and ‘Jimai’, ‘Shimai’, ‘Hanmai’, and ‘Liangxing 99’ lines are the main wheat cultivars in Hebei. Therefore, the difference in wheat cultivars in these regions may result in different selection pressures on *P. triticina* populations, which may cause the virulence or dominant races of *P. triticina* to evolve in different directions and may also easily lead to the diversity of *P. triticina* races, resulting in the changes in population structure. Moreover, precipitations, temperatures, and altitude can affect the development of *P. triticina* populations [50]. The three wheat-growing regions (including the four provinces) have different geographical and climatic conditions. Shandong and Hebei belong to the temperate monsoon climate, Sichuan belongs to the subtropical monsoon climate and plateau mountain climate, and Xinjiang belongs to the temperate continental climate. This significant climatic difference in these three wheat-growing regions may affect the fitness of *P. triticina*, the size of *P. triticina* populations, and the composition of dominant races, and may then affect the *P. triticina* population structure.

Migration was also an important factor in the genetic structure of *P. triticina* populations [51,52,53]. The spores of *P. triticina* can spread long distances with airflow, which results in gene flow among populations in different regions, and gene flow could change the gene frequency of the receiving population. We found a relatively high level of gene flow (*Nm* = 2.82 > 1) among the four populations by using the AMOVA method of Arlequin3.ll software. *Nm* (gene flow) > 1 means gene flow between populations, which could prevent genetic differentiation between populations [54]. In this study, we found a significant correlation between genetic polymorphism and the geographical origin of four populations, and the populations of Hebei, Shandong, and Sichuan had a closer genetic relationship, while the Xinjiang population was a relatively independent population. This may be related to the geographical environment of the four provinces. Hebei and Shandong are mainly affected by the southeast monsoon from the Pacific Ocean, and the two provinces are adjacent to each other, so *P. triticina* isolates can easily spread to Hebei from Shandong. The southwest monsoon in the southwest region of China, emanating from the Indian Ocean, affects Yunnan, Chongqing, Sichuan, and even Southeast China. So, *P. triticina* from India may migrate to China along with the monsoon, which may be the reason that there are similar predominant races in China and India; THTT and PHTT are the predominant races in both regions [22]. Moreover, according to the occurrence and development time of leaf rust in the four provinces, the peak occurrence time of leaf rust in Sichuan is from March to the end of April, in Shandong from mid May to early June, and in Hebei, it is a few days later than that in Shandong. Therefore, the *P. triticina* isolates in the southwest wheat-growing region are likely to spread towards southeast China with the airflow of southwest wind in May, then spread to Shandong and then Hebei by the southeast wind, resulting in gene flow among the three regions. Compared with the other three provinces, the Xinjiang region is located in the northwest of China and has a relatively high altitude. This region is affected by the west monsoon from the Atlantic Ocean. Hence, *P. triticina* may spread from west to east along the Tianshan Mountains in Xinjiang. Moreover, leaf rust in the western region of Xinjiang occurs earlier and is more severe than that in the eastern region. However, the wheat-growing regions in Xinjiang are geographically isolated, separated at a long distance from other wheat-growing regions of China by deserts and mountains [33], so the migration of *P. triticina* isolates is more difficult and most isolates may only spread within Xinjiang like *P. striiformis* [55], although there was some gene flow between Xinjiang and the other three provinces. Therefore, the above factors led to higher genetic similarity among the *P. triticina* populations of Hebei, Shandong, and Sichuan, while the *P. triticina* populations of Xinjiang were relatively independent.

This study found no significant correlation between virulence phenotypes and genotypes of the four *P. triticina* populations. This was consistent with the results of other studies on the population diversity of *P. triticina* in China [56,57]. For example, our previous study found no significant correlation between virulence and molecular polymorphism of *P. triticina* populations in Hebei Province of China from 2001 to 2010 [58]. Similar results were reported in Western Europe, South Asia, Russia, and Kazakhstan [35,59,60]. In addition, the same conclusion had been reported in studies on the population genetics of *P. striiformis* f. sp. *tritici* in the United States and Northwestern China [33,61]. However, there were also some different views on the correlation between virulence phenotypes and molecular genotypes of *P. triticina*. Some studies on genetic diversity have reported a significant correlation between virulence phenotypes and molecular genotypes of *P. triticina* in Pakistan, Europe, Central Asia, the Caucasus regions, North America, South America, and other worldwide regions [31,39,44,52,62,63,64,65,66,67,68,69], which was controversial with the views of the above studies.

The polymorphism analysis of 101 *P. triticina* isolates of four predominant pathotypes showed a significant correlation between pathotype and virulence polymorphism, not between phenotype and EST-SSR polymorphism. The majority of the same pathotypes were grouped into one cluster or the same subcluster based on virulence, although the clustering of individual pathotypes had a certain correlation with the regional origin. In the EST-SSR polymorphism analysis, different pathotypes were clustered into one cluster or subcluster, but these pathotypes had a certain correlation with their regional origin. These results indicated that the same pathotypes did not completely cluster into one group. In contrast, the different pathotypes may be clustered, revealing that these isolates were still different or identical although they were of the same or different pathotypes, which also showed that the virulence phenotype was not correlated with the molecular genotype. Similar results have also been reported by Wang et al. [42], who noted that TDBG and TDBJ, which differed in virulence on *Lr14a*, were clustered in the same cluster. The similarity of virulence might be due to the selection effect of wheat cultivars with different leaf rust resistance genes. Zhao [70] and Wang [71] thought that there was virulence heterogeneity in the same pathotype of *P. triticina*, and the pathogenicity of different isolates of the same pathotype of *P. triticina* was not completely consistent.

## 4. Materials and Methods

### 4.1. Leaf Rust Sample Collection and Multiplication

Leaf rust samples were collected from Heibei Province, Shandong Province, Sichuan Province, and the Xinjiang Uygur Autonomous Region in 2009 in China by researchers from the College of Plant Protection, Hebei Agricultural University (Table 1). All samples were from fields under natural infection. Wheat leaves with uredinia of *P. triticina* collected from a single plant or cultivar were treated as a single sample. The sample treatment and single-uredinial isolates multiplication were performed as described by Zhang et al. [7]. Urediniospores collected from each sample were inoculated to the susceptible cultivar ‘Zhengzhou 5389’ to obtain the single uredinium. Urediniospores from the single uredinium were increased by inoculating the new seedlings of ‘Zhengzhou 5389’ using the same inoculation procedure. Twelve days after inoculation, urediniospores were collected by shaking the leaf and putting the spores into glass tubes to dry, after which they were lyophilized and stored at −20 °C.

### 4.2. Race and Virulence Phenotype Data

Race identification and virulence determination of isolates was performed as described by Zhang et al. [7]. Urediniospores collected from each sample were inoculated on two sets of Thatcher near-isogenic lines, which including 16 standardized differentials (with single resistance genes *Lr1*, *Lr2a*, *Lr2c*, *Lr3*; *Lr9*, *Lr16*, *Lr24*, *Lr26*; *Lr3Ka*, *Lr11*, *Lr17*, *Lr30*; *LrB*, *Lr10*, *Lr14a*, and *Lr18*) and 18 extra differentials (with resistance genes *Lr2b*, *Lr3bg*, *Lr14b*, *Lr15*, *Lr19*, *Lr21*, *Lr23*, *Lr28*, *Lr33*, *Lr33 + 34*, *Lr36*, *Lr37*, *Lr38*, *Lr39*, *Lr42*, *Lr44*, *Lr47,* and *Lr50*). The susceptible Thatcher was used as a control. When uredinia were fully developed on the leaves of Thatcher about 12 d post-inoculation (dpi), infection types (IT) on the near-isogenic lines were recorded based on a 0 to 4 scale [72]. Races were determined according to Kolmer [38].

### 4.3. DNA Extraction and PCR Amplification

The genomic DNA of each isolate sample was extracted from the urediniospores according to the modified CTAB method described by Gao [73]. The 21 pairs of EST-SSR primers developed by Wang were used for PCR reaction [43], and the total reaction system and amplification procedure were performed as described by Zhang et al. [40]. PCR products were separated by 10% (*w*/*v*) polyacrylamide gel in a 0.5 × TBE buffer (45 mM Tris, 45 mM Boric acid, and 1 mM EDTA) and were visualized by silver staining.

### 4.4. Data Analysis

To analyze the genetic relationships among the selected *P. triticina* isolates, two binary matrices based on virulence infection types (IT) and EST-SSR data were constructed, respectively, as described by Zhang et al. [40]. The software program NTSYS-pc version 2.10 [74] was used to construct the dendrograms, the 2D principal coordinate analysis, and the correlation analysis between the virulence and EST-SSR. The software POPGENE version 1.32 [75] was used to assess the population diversity of *P. triticina*. To evaluate population genetic differentiations, an analysis of molecular variance (AMOVA) was performed using the AMOVA model of the Arlequin ver. 3.11 software.

## Figures and Tables

**Table 1:** Isolate codes, location, and race information of *Puccinia triticina* samples collected from Hebei, Shandong, Sichuan, and Xinjiang.

Isolate Codes	Region	Races	Isolate Codes	Region	Races	Isolate Codes	Region	Races
1	Tangshan, Hebei	THTS	74	Yantai, Shandong	PHKT	147	Shihezi, Xinjiang	THKT
2	Tangshan, Hebei	THTT	75	Yantai, Shandong	THTT	148	Urumqi, Xinjiang	THPT
3	Tangshan, Hebei	PHHP	76	Jinan, Shandong	PHNS	149	Urumqi, Xinjiang	TCPT
4	Tangshan, Hebei	THTS	77	Jinan, Shandong	PHKT	150	Urumqi, Xinjiang	PCTS
5	Tangshan, Hebei	PHST	78	Jinan, Shandong	PHKT	151	Urumqi, Xinjiang	MRTT
6	Tangshan, Hebei	THTS	79	Jinan, Shandong	THKT	152	Urumqi, Xinjiang	THTT
7	Tangshan, Hebei	PHJT	80	Jinan, Shandong	THKS	153	Urumqi, Xinjiang	THTT
8	Tangshan, Hebei	PHTT	81	Jinan, Shandong	PHKT	154	Kuitun, Xinjiang	TCRF
9	Qinhuangdao, Hebei	FHTT	82	Jinan, Shandong	PHKT	155	Kuitun, Xinjiang	PRTT
10	Qinhuangdao, Hebei	RHTT	83	Jinan, Shandong	PHKH	156	Kuitun, Xinjiang	PGPT
11	Qinhuangdao, Hebei	PHKS	84	Jinan, Shandong	THTT	157	Kuitun, Xinjiang	THTS
12	Qinhuangdao, Hebei	PGTQ	85	Jinan, Shandong	PHTT	158	Urumqi, Xinjiang	MCPS
13	Langfang, Hebei	THTS	86	Yantai, Shandong	THTT	159	Urumqi, Xinjiang	MHQS
14	Langfang, Hebei	PHTT	87	Yantai, Shandong	THTT	160	Shihezi, Xinjiang	PHTT
15	Cangzhou, Hebei	THPT	88	Laiyang, Shandong	THKS	161	Yining, Xinjiang	TRRT
16	Cangzhou, Hebei	THKT	89	Tai’an, Shandong	THTT	162	Yining, Xinjiang	THTS
17	Cangzhou, Hebei	THTS	90	Laiyang, Shandong	PHKR	163	Yining, Xinjiang	TCTT
18	Baoding, Hebei	PHBT	91	Laiyang, Shandong	PHTS	164	Shihezi, Xinjiang	THTT
19	Baoding, Hebei	TGKT	92	Laiyang, Shandong	THTS	165	Shihezi, Xinjiang	TGTS
20	Baoding, Hebei	PHKT	93	Laiyang, Shandong	THTT	166	Shihezi, Xinjiang	PHNS
21	Baoding, Hebei	PHST	94	Laiyang, Shandong	PHKT	167	Urumqi, Xinjiang	THTT
22	Baoding, Hebei	THTT	95	Laiyang, Shandong	PHSS	168	Shihezi, Xinjiang	TKFS
23	Baoding, Hebei	THTS	96	Yantai, Shandong	THTT	169	Urumqi, Xinjiang	PHTS
24	Shijiazhuang, Hebei	TKTR	97	Jiange, Sichuan	THRT	170	Yining, Xinjiang	THTS
25	Handan, Hebei	THKT	98	Jiange, Sichuan	PHFS	171	Shihezi, Xinjiang	PHTT
26	Handan, Hebei	THTS	99	Jiange, Sichuan	TCTT	172	Urumqi, Xinjiang	THTT
27	Handan, Hebei	THTT	100	Jiange, Sichuan	PHTT	173	Shihezi, Xinjiang	PHRT
28	Handan, Hebei	THGT	101	Jiange, Sichuan	THTT	174	Shihezi, Xinjiang	CHKT
29	Handan, Hebei	THHS	102	Jiange, Sichuan	PHTT	175	Yining, Xinjiang	THPP
30	Xingtai, Hebei	THTT	103	Jiange, Sichuan	THST	176	Shihezi, Xinjiang	FHTT
31	Tangshan, Hebei	PHTT	104	Jiange, Sichuan	THTT	177	Shihezi, Xinjiang	THTS
32	Tangshan, Hebei	PHKS	105	Jiange, Sichuan	PHTS	178	Shihezi, Xinjiang	TCTM
33	Tangshan, Hebei	PHTT	106	Jiange, Sichuan	THTT	179	Shihezi, Xinjiang	THTT
34	Tangshan, Hebei	THKT	107	Jiange, Sichuan	TKFS	180	Shihezi, Xinjiang	PHRT
35	Langfang, Hebei	PHTS	108	Zitong, Sichuan	TCTT	181	Urumqi, Xinjiang	PHTT
36	Shijiazhuang, Hebei	PHKQ	109	Zitong, Sichuan	THTT	182	Yining, Xinjiang	THTP
37	Shijiazhuang, Hebei	THTT	110	Panzhihua, Sichuan	THKT	183	Yining, Xinjiang	THPR
38	Shijiazhuang, Hebei	PHNQ	111	Panzhihua, Sichuan	THTT	184	Shihezi, Xinjiang	TCTT
39	Shijiazhuang, Hebei	THTT	112	Panzhihua, Sichuan	FHTT	185	Urumqi, Xinjiang	THSR
40	Handan, Hebei	THTT	113	Panzhihua, Sichuan	PHGT	186	Yining, Xinjiang	THTT
41	Handan, Hebei	PHKS	114	Ya’an, Sichuan	TGJP	187	Shihezi, Xinjiang	TCKT
42	Xingtai, Hebei	THTT	115	Yibin, Sichuan	TKKD	188	Shihezi, Xinjiang	THTT
43	Xingtai, Hebei	THSS	116	Zitong, Sichuan	PHTT	189	Shihezi, Xinjiang	THTS
44	Tangshan, Hebei	THTT	117	Panzhihua, Sichuan	TKTT	190	Kuitun, Xinjiang	PHTS
45	Tangshan, Hebei	PHTQ	118	Jiange, Sichuan	PHFS	191	Yining, Xinjiang	THSR
46	Handan, Hebei	THSS	119	Jiange, Sichuan	THTT	192	Shihezi, Xinjiang	THTT
47	Langfang, Hebei	PHGS	120	Jiange, Sichuan	PKPQ	193	Urumqi, Xinjiang	THRR
48	Langfang, Hebei	THTT	121	Jiange, Sichuan	THTS	194	Shihezi, Xinjiang	THTT
49	Tai’an, Shandong	PCJT	122	Jiange, Sichuan	PHKN	195	Kuitun, Xinjiang	THKQ
50	Tai’an, Shandong	PHKT	123	Zitong, Sichuan	FHTR	196	Yining, Xinjiang	THTS
51	Tai’an, Shandong	THTS	124	Zitong, Sichuan	THTT	197	Urumqi, Xinjiang	THSR
52	Tai’an, Shandong	PHTT	125	Zitong, Sichuan	THTS	198	Shihezi, Xinjiang	PHTT
53	Laiyang, Shandong	PCHT	126	Panzhihua, Sichuan	TKTQ	199	Yining, Xinjiang	THPN
54	Laiyang, Shandong	THTT	127	Panzhihua, Sichuan	PHTP	200	Kuitun, Xinjiang	PGST
55	Laiyang, Shandong	PHKS	128	Yibin, Sichuan	THTT	201	Kuitun, Xinjiang	THTT
56	Laiyang, Shandong	THTT	129	Yibin, Sichuan	THJT	202	Shihezi, Xinjiang	TGTQ
57	Yantai, Shandong	PHKT	130	Yibin, Sichuan	THTT	203	Urumqi, Xinjiang	THTT
58	Yantai, Shandong	THRT	131	Yibin, Sichuan	THTJ	204	Yining, Xinjiang	KHPQ
59	Yantai, Shandong	PHRT	132	Jiange, Sichuan	FCKT	205	Kuitun, Xinjiang	TBTT
60	Qingdao, Shandong	PHTT	133	Jiange, Sichuan	FHRT	206	Yining, Xinjiang	PHKQ
61	Laiyang, Shandong	PHKT	134	Jiange, Sichuan	THKP	207	Shihezi, Xinjiang	THTT
62	Laiyang, Shandong	THTT	135	Panzhihua, Sichuan	PHST	208	Urumqi, Xinjiang	THTT
63	Laiyang, Shandong	THTT	136	Jiange, Sichuan	THTT	209	Urumqi, Xinjiang	TGTP
64	Laiyang, Shandong	PHRS	137	Jiange, Sichuan	THTT	210	Shihezi, Xinjiang	THTT
65	Laiyang, Shandong	THRS	138	Zitong, Sichuan	PHTT	211	Yining, Xinjiang	PGDL
66	Laiyang, Shandong	PHKT	139	Panzhihua, Sichuan	PHST	212	Shihezi, Xinjiang	PHTS
67	Yantai, Shandong	PHKT	140	Panzhihua, Sichuan	PHTT	213	Shihezi, Xinjiang	PHTT
68	Yantai, Shandong	THKT	141	Shihezi, Xinjiang	THTP	214	Shihezi, Xinjiang	THTS
69	Yantai, Shandong	PHTT	142	Shihezi, Xinjiang	MGRR	215	Yining, Xinjiang	PHTT
70	Yantai, Shandong	THTT	143	Yining, Xinjiang	THTT	216	Urumqi, Xinjiang	PHRM
71	Yantai, Shandong	THTT	144	Yining, Xinjiang	THTP	217	Yining, Xinjiang	THTP
72	Yantai, Shandong	THTT	145	Yining, Xinjiang	PHTT			
73	Yantai, Shandong	THHT	146	Yining, Xinjiang	TCCT			

**Table 2:** Races and number of isolates determined from Hebei, Shandong, Sichuan, and Xinjiang in 2009.

Regions	Number of Isolates	Number of Races	Races
Hebei	48	24	**THTT**(10), **THTS**(7), **PHTT**(4), **PHJS**(3), **THJT**(3), PHST(2), THJS(2), PHGP, FHTT, RHTT, PGTQ, THPT, PHBT, TGJT, PHKT, PHJT, TKTR, THGT, THGS, PHTS, PHJQ, PHPQ, PHTQ, PHGS
Shandong	48	19	**THTT**(14), **PHJT**(11), **PHTT**(4), THJS(2), THJT(2), THTS(2), PCJT, PCGT, PHJS, THRT, PHRT, PHRS, THRS, THGT, PHPS, PHJH, PHJR, PHTS, PHSS
Sichuan	44	26	**THTT**(11), **PHTT**(5), **THJT**(3), TCTT(2), THTS(2), PHDS(2), PHST(2), THRT, PHTS, TKDS, FHTT, PHGT, TGJP, TKJD, TKTT, PKPQ, PHJN, FHTR, TKTQ, PHTP, THTJ, FCJT, FHRT, THJP
Xinjiang	77	41	**THTT**(16), **THTS**(7), **PHTT**(7), **THTP**(4), THTR(3), PHTS(3), TCTT(2), PHRT(2), MGRR, TCBT, THJT, THPT, TCPT, PCTS, MRTT, TCRF, PRTT, PGPT, MCPS, MHRS, TRRT, TGTS, PHPS, TKDS, CHJT, THPP, FHTT, TCTM, THPR, TCJT, THRR, THJQ, THPN, PGTT, TGTQ, KHPQ, TBTT, PHJQ, TGTP, PGDL, PHRM

Note: The number in parentheses is for the number of races, while races without parentheses only have one isolate; bold is for the predominant races in each region.

**Table 3:** Distribution and frequencies of predominant races of *Puccinia triticina* among single uredinal isolates taken from leaf rust samples collected from wheat fields from Hebei, Shandong, Sichuan, and Xinjiang.

Races	Hebei		Shandong		Sichuan		Xinjiang		Total	
	N	%	N	%	N	%	N	%	N	%
THTT	10	20.83	14	29.17	11	25.00	16	20.78	51	23.50
PHTT	4	8.33	4	8.33	5	11.36	7	9.09	20	9.22
THTS	7	14.58	2	4.17	2	4.55	7	9.09	18	8.29
PHKT	1	2.08	11	22.92	0	0.00	0	0.00	12	5.53
THKT	3	6.25	2	4.17	1	2.27	1	1.30	7	3.23
PHTS	1	2.08	1	2.08	1	2.27	3	3.90	6	2.76
PHKS	3	6.25	1	2.08	0	0.00	0	0.00	4	1.84
PHST	2	4.17	0	0.00	2	4.55	0	0.00	4	1.84
TCTT	0	0.00	0	0.00	2	4.55	2	2.60	4	1.84
THTP	0	0.00	0	0.00	0	0.00	4	5.19	4	1.84
FHTT	1	2.08	0	0.00	1	2.27	1	1.30	3	1.38
PHRT	0	0.00	1	2.08	0	0.00	2	2.60	3	1.38
THSR	0	0.00	0	0.00	0	0.00	3	3.90	3	1.38
others	16	33.33	12	25.00	19	43.18	31	40.26	78	35.94
Total	48	...	48	...	44	...	77	...	217	...

**Table 4:** Numbers of alleles, polymorphic alleles, and percent of polymorphism of 18 EST-SSR primers.

EST-SSR Locus	Number of Alleles ^a^	Number of Polymorphic Alleles ^b^	Percent of Polymorphic (%)	EST-SSR Locus	Number of Alleles ^a^	Number of Polymorphic Alleles ^b^	Percent of Polymorphic (%)
Ptssr0083	2	1	50.00	Ptssr0189	4	2	50.00
Ptssr0019	2	1	50.00	Ptssr0801	2	1	50.00
Ptssr5649	3	2	66.67	Ptssr0481	3	2	66.67
Ptssr0085	3	2	66.67	Ptssr0639	3	2	66.67
Ptssr2948	3	2	66.67	Ptssr3145	2	1	50.00
Ptssr0536	2	1	50.00	Ptssr6542	3	3	100.00
Ptssr6863	2	1	50.00	Ptssr0182	2	1	50.00
Ptssr0243	2	1	50.00	Ptssr6386	4	3	75.00
Ptssr0125	3	2	66.67	Average	2.67	1.67	62.50
Ptssr5594	3	2	66.67	Total	48	30	62.50

^a^ The maximum number of alleles each pair of primers can amplify. It should be noted that not every isolate can amplify all alleles. ^b^ The number of polymorphic alleles contained in the above-mentioned alleles (alleles that cannot be amplified in certain isolates).

**Table 5:** Genetic diversity parameters of *Puccinia triticina* based on EST-SSR.

Genetic Polymorphic Parameters	Hebei	Shandong	Sichuan	Xinjiang	Species Level	Populations Level
Observed number of alleles (*Na*)	1.84	1.67	1.71	1.82	1.95	1.76
Effective number of alleles (*Ne*)	1.67	1.50	1.49	1.49	1.62	1.54
Nei’s gene diversity index (*H*)	0.36	0.28	0.28	0.28	0.35	0.30
Shannon’s information index (*I*)	0.52	0.40	0.41	0.42	0.52	0.40
Number of polymorphic loci (*NP*)	41.00	33.00	35.00	40.00	47.00	37.00
Percent of polymorphic loci (*P*%)	83.67	67.35	71.43	81.63	95.92	76.02

**Table 6:** Analysis of molecular variance (AMOVA) across and within populations.

Source of Variation	Degree of Freedom	Sum of Squares	Percentage of Variation	*p* Value
Among populations	3	306.14	25.03	<0.0001
Within populations	213	1159.06	74.97	<0.0001
Total	216	1465.20	100.00	

**Table 7:** Nei’s unbiased measures of genetic distances and genetic identities between populations.

Populations	Hebei	Shandong	Sichuan	Xinjiang
Hebei	****	0.9337	0.9318	0.9229
Shandong	0.0686	****	0.9089	0.8587
Sichuan	0.0707	0.0955	****	0.8682
Xinjiang	0.0803	0.1524	0.1413	****

Note: Nei’s genetic identities (above diagonal) and genetic distances (below diagonal).

## Data Availability

The data presented in this study are available upon request from the corresponding author.
